# Preliminary results for the use of proteinase K to achieve release of LPS from the Alteco LPS Adsorber^® ^after perfusion with LPS containing blood

**DOI:** 10.1186/cc11782

**Published:** 2012-11-14

**Authors:** E Hansen, L Pierre, S Blomqvist

**Affiliations:** 1 Alteco Medical AB, Lund, Sweden; 2 Faculty of Medicine, Lund University, Sweden

## Background

The effect of Alteco LPS Adsorber^® ^to remove LPS from the circulation is based on the incorporation of a synthetic peptide that binds to the lipid A moiety of LPS, the binding capacity in one adsorber exceeding 7.5 μg LPS. Positive clinical effects of the adsorber when used in patients with Gram-negative sepsis have been reported [1]. Measurement of LPS in human blood, however, is hampered by difficulties such as contamination and interference. The aim of this pilot study was to evaluate a method to release LPS from the LPS adsorber after perfusion with LPS containing blood.

## Methods

Two adsorbers (A1, A2) containing the active peptide and one dummy adsorber (D) with no peptide were used. Whole blood (500 ml) from healthy pigs was collected in a bag containing heparin, 37 μg LPS was added. A roller pump was used to recirculate the blood through the adsorber. The pump flow was set at 150 ml/minute and the duration of the perfusion was 2 hours. After the end of perfusion the adsorbers were rinsed with 500 ml Krebs solution. A solution of 20 mg proteinase K in 50 ml Tris buffer with pH 7.4 was prepared and 2.5 ml CaCl_2 _was added. The adsorbers were kept at 37°C and the proteinase K solution [2] was perfused through the adsorbers at 5 ml/minute for 6 hours. Samples for LPS analysis (chromogenic LAL) [3] were drawn before the perfusion and then after 30, 240 and 360 minutes. The enzyme activity was checked using a synthetic substrate [4].

## Results

The concentrations of LPS before and during perfusion with proteinase K are shown in Table 1.

**Table 1:** 

LPS	Before	30 minutes	240 minutes	360 minutes
A1 (EU/ml)	3.65	5.29	27.04	50.68
A2 (EU/ml)	2.74	4.65	10.45	18.04
D (EU/ml)	3.94	4.16	6.78	5.82
Enzyme activity (%), A1, A2, D	100, 100, 100	104, 110, 110	96, 102, 104	92, 95, 102

## Conclusion

The LPS values found before the start of the perfusion indicate contamination of the solution. The increase in LPS seen in all adsorbers after 30 minutes is probably due to traces of blood components. The later increase in LPS after treatment with proteinase K in the active adsorbers indicates that the adsorber is effective in capturing LPS from whole blood and that proteinase K is able to dislodge LPS bound to the adsorber.
